# Genome Sequence of a New Siphoviridae Phage Found in a Brazilian Bacillus thuringiensis Serovar israelensis Strain

**DOI:** 10.1128/genomeA.01606-17

**Published:** 2018-05-31

**Authors:** Fabrício S. Campos, Gil R. Santos, Vitor L. Nascimento, Roberto F. T. Corrêia, Alex S. R. Cangussu, Karen Hoffmann, Eugênio E. Oliveira, Bergmann M. Ribeiro, Raimundo W. S. Aguiar

**Affiliations:** a Biotechnology Graduate Program, Federal University of Tocantins, Gurupi, Tocantins, Brazil; b Plant Production Graduate Program, Federal University of Tocantins, Gurupi, Tocantins, Brazil; c Entomology Department, Federal University of Viçosa, Viçosa, Minas Gerais, Brazil; d Molecule Biology Graduate Program, University of Brasilia, Brasilia, Brazil; e Biotechnology and Plant Production Graduate Program, Federal University of Tocantins, Gurupi, Tocantins, Brazil

## Abstract

During the fermentation process, Bacillus thuringiensis (Bt) phages can result in bacterial death and decreased yield. In this work, we describe the genome of a new phage related to the Siphoviridae viral family from a Brazilian strain of Bt which showed high nucleotide sequence identity to the genomes of phages phi4l1 and BtCS33.

## GENOME ANNOUNCEMENT

Bacillus thuringiensis (Bt), a Gram-positive bacterium, has been used for biocontrol of insect pests for many years (1, 2). About 83% of Bt strains have lysogenic phage inserted in their genome (3). During Bt fermentation, lysogenic phages can cause bacterial death in 15% to 30% of the batches, resulting in decreased Bt yields (4). However, the exact mechanism involved in the activation of the phage lytic cycle is still unclear. In order to understand the lysogeny control mechanism and reduce losses during Bt fermentation, more genetic information from Bt phages is needed (3).

In this work, we report the identification of a lysogenic phage genome from a Brazilian B. thuringiensis serovar israelensis strain called BtiUFT6.51-F, which was shown to be highly toxic to Lepidoptera larvae (data not shown). This new bacteriophage was identified by sequencing the full genome of BtiUFT6.51-F. Briefly, the phage genome was identified by the reads obtained with total DNA of BtiUFT6.51-F sequencing using the Geneious program's Map to Reference tool with Bacillus phage phi4Ii as a reference. The phage was shown to have high nucleotide sequence identities to the phi4l1 (99.8%) and BtCS33 (99.3%) phage genomes from the Siphoviridae family previously described in Bt strains. The small differences in the genome sequence were found in phage tail genes. Total DNA extraction was carried out using 200 μl of the bacterial suspension, as described elsewhere (5). Sequencing was performed by next-generation sequencing (NGS) in a MiSeq (Illumina) platform at the Catholic University of Brasília, Brasília, Distrito Federal, Brazil. A total of 98,608 reads were generated, with an average length of 77 bp, i.e., a coverage of 180× the whole phage genome. Reads were trimmed and assembled (Geneious 9.0 software [6]) into a single contig of 42,076 bp. This new virus genome sequence was called BtiUFT6.51-F and showed 35.3% GC content and 63 open reading frames (ORFs). Like other Bt phages (BtCS33 isolated from Bacillus thuringiensis serovar kurstaki strain CS-33), the BtiUFT6.51-F genome is organized in three main function-related gene clusters: the late region (encoding structural, assembly, DNA packaging, and lysis proteins), the lysogeny-lysis control region (encoding proteins for controlling the lysogeny-lysis process), and the early region (encoding proteins for phage DNA replication, recombination, and modification). The high similarity between phages from different regions and countries indicates a close evolutionary relationship among Bacillus species.

### Accession number(s).

The genome sequence of BtiUFT6.51-F was deposited under GenBank accession number MG710484.
